# Evaluation of the diagnostic implications of Das-Naglieri cognitive assessment system in children with attention deficit hyperactivity disorder

**DOI:** 10.1186/s12888-018-1970-x

**Published:** 2018-12-12

**Authors:** Ling Qin, Hairun Liu, Hong Zhang, Yanhong Fu, Dandan Li

**Affiliations:** From the center of Cognitive and Sleeping, The people’s hospital of Guangxi Zhuang autonomous region, Nanning, 530021 Guangxi China

**Keywords:** Attention deficit hyperactivity disorder (ADHD), Das-Naglieri cognitive assessment system (DN:CAS), PASS, Cognitive assessment

## Abstract

**Background:**

Attention deficit hyperactivity disorder (ADHD) is a neurodevelopmental characterized by attention deficit, hyperactivity, emotional impulses and difficulty with cognitive functions. The Das-Naglieri Cognitive Assessment System (DN: CAS), as a theory-driven assessment kit, was explored based on Planning, Attention, Simultaneous, and Successive Theory (PASS). Recent researches have tried to explore the sensitivity and specificity of DN: CAS in diagnosing ADHD; nevertheless, these studies were performed in a small study population. The following study explores the cognitive functions in ADHD by the DN: CAS and to evaluate the DN: CAS’s diagnostic value in ADHD.

**Methods:**

A total of 135 children with ADHD and 140 healthy controls were enrolled to evaluate cognitive function by the DN: CAS. ROC curve and the area under the curve (AUC) were applied to evaluate the diagnostic value of DN: CAS on ADHD.

**Results:**

Compared with healthy controls, children with ADHD had significantly lower scores in Planning, Simultaneous (Verbal-Spatial Relations), Attention in the four Subtests of DN: CAS, as well as the total scores. ROC analysis indicated that Planning and Attention of DN: CAS had good classification accuracy in diagnosing ADHD with AUCs of 0.808 and 0.730, respectively.

**Conclusions:**

The planning and attention assessment of DN: CAS revealed high sensitivity and specificity in diagnosing ADHD, thus suggesting that DN: CAS might be an effective tool in diagnosing ADHD.

## Background

In China, approximately 4.31–5.83% of children are affected by ADHD, which is a neurodevelopmental disorder characterized by attention deficit, hyperactivity, emotional impulses and difficulty with cognitive functions [1, 2]. The American Academy of Child & Adolescent Psychiatry noted that the assessments for ADHD may include structured diagnostic interviews, parent and teacher report behavior rating scales, direct observations by doctor and cognitive tests. As cognitive neuroscience advances, most studies seem to demonstrate that the impairment of cognitive function is an important manifestation in ADHD [3–5]. Barkley constructed a theoretical model indicating that ADHD may be linked to four executive neuropsychological function impairments in 1997 [6]. These theories provided the basis for clinical diagnosis of ADHD and many cognitive assessment tools were used to evaluate the clinical symptoms of ADHD such as CPT test, Stroop test and Go/No Go task [7–10].

The Das-Naglieri cognitive assessment system (DN: CAS), as a theory-driven assessment kit, was explored based on PASS theory by JP. Das, which was applied to assess the cognitive function. PASS theory has reconceptualized intelligence as a process-driven understanding of cognitive abilities based on the following four cognitive processes: Planning, Attention, Simultaneous and Successive processing [11]. This theory has been linked to Luria’s three functional units of the brain. Attention has been linked to the first structural unit, i.e. brain stem, midbrain and the brain; Simultaneous and Successive processing have been linked to the second structural unit, i.e. occipital, temporal, and parietal lobes; Planning has been linked with the third structural unit, i.e. the frontal lobe, and especially the prefrontal lobe in the same direction [12]. In his study, McCrea has reported on three patients with unilateral focalized stroke lesions that were examined longitudinally on the DN: CAS, suggesting that the DN: CAS subtests are not only unique but also sensitive and specific to focalized cortical lesions [13].

PASS theory, which is similar to Barkley’s execution function defect theory, proposes that children with ADHD may be more impulsive in cognitive processing, which in turn can influence planning processing. Attention difficulties are expected to negatively affect attention processing. Some studies have demonstrated that children with ADHD assessed by DN: CAS have the lowest performance on Planning and Attention scores, whereas their Simultaneous and Successive processing scores appear normal [14, 15]. DN: CAS has been standardized in different languages including Chinese [16].

ADHD is a multifactorial disorder, and genetic factors might be associated with the risk of ADHD [17, 18]. Given the different genetic background, the aim of the present study was to explore the association of cognitive functions and ADHD, and to evaluate the DN: CAS’s diagnostic value in Chinese children with ADHD.

## Methods

### Study population

A total of 135 children with ADHD and 140 controls were recruited in this study. The ADHD children were enrolled from People’s hospital of Guangxi Zhuang Autonomous Region between Jan 2015 and Dec 2016. In addition, 140 healthy controls were enrolled from the same communities and schools. All study subjects were interviewed by two well-trained investigators at the Department of Psychiatry, who used K-SADS to conduct interview. ADHD was diagnosed according to American Psychiatric Association (APA) criteria (Diagnostic and statistical manual of mental disorders, 5th edition (DSM-5)). Medical history, clinical examination and APA diagnosis criteria evaluated by two independent psychiatrists were used to identify healthy controls as free of ADHD. Children with Intellectual Disability (The Full Scale IQ (FSIQ) < 85 by WISC-CR-II), Learning Disorder, Tic Disorders and Autism spectrum disorder were excluded from the study. The FSIQ was included in the present study.

The Ethics Committee of People’s hospital of Guangxi Zhuang autonomous Region approved this study, and all study subjects agreed to participate in our research after they were debriefed about the study and after they signed the informed consent; for the children under 16-years of age, the consent was obtained from their guardians.

### Instrument

#### DN:CAS Chinese version

The DN: CAS [16] was administered by a well-trained psychotherapist. This assessment tool includes four subscales, including Planning, Attention, Simultaneous Processing, and Successive Processing. The whole test is made up of 12 subtests, with each subscale containing three items. Two different sets of tests were carried out according to various age groups (5–7 year-olds and 8–17 year-olds).

Planning assessment was used to evaluate the efficiency in task solving, including Matching Numbers, Planned Codes and Planned Connections. Matching Numbers requires the test taker to find two identical numbers in the same row (e.g. find 2 identical numbers in a row such as 3–5–2-6-5-7-4). Arrangement in each row is well-designed in order to detect the benefit of the matching strategy during differentiation. Planned Codes require the test taker to code characters arranged in specific order with certain strategy, based on the way they’ve been previously thought (e.g. Codes for A, B and C are OX, XX, and XO, separately. The subject needs to convert a series of characters into corresponding codes). Planned Connections require the subject to connect randomly scattered numbers/numbers and letters in numerical or/and alphabetical order (e.g. 1–2–3-4/1-A-2-B-3-C).

Attention mainly assesses the ability to selectively focus on one part of the two-dimensional stimulus while ignoring the other part, including Expressive Attention, Number Detection and Receptive Attention. Expressive Attention refers to the ability to inhibit interfering stimuli in the procedure of expression (e.g. test taker is presented with cards containing the color names printed in colors that are different from the meaning of the printed word, and then the test taker is asked to say the color of the word instead of the word itself). Number Detection evaluates selective attention, switching attention and cognitive inhibition to distraction (e.g. test taker is presented with 3–6 targeted numbers together with several rows of numbers from 0 to 9 in the item, and then he/she is asked to find and underline figures that look exactly the same as targeted ones, from left to right in a row). Receptive Attention requires the subject to circle every pair of characters that look the same or that have the same pronunciation (e.g. circle AA but not AB, and circle Tt but not Tb).

Simultaneous processing requires the test taker to interconnect components of special items to get the correct answer. It includes Nonverbal Matrices, Verbal-Spatial Relations and Figure Memory. The test taker needs to figure out relation among the segments in the item, then to integrate them with abstract thinking and logical perception ability. There are three types of items in Nonverbal Matrices, i.e. graph filling, analogical reasoning and spatial vision. Each item contains graphs and geometrical elements with relations to either spatial organization or logical organization, and the subject reveals abstract reasoning by observing these relations (e.g. he/ she is asked to make a deduction and find out the absent figure based on the relationship among the five figures presented). In Verbal-Spatial Relations, the subject needs to choose a corresponding picture after he/she is given the description with underlying logical relationship (e.g. subject is presented with six pictures showing various spatial relationships between a ball and a desk, and asked to point out the correct picture). Figure Memory is a representative subtest of simultaneous processing. It includes two-dimensional or three-dimensional figures that are shown to subject, who is then asked to discriminate the simple figure from a complex one (e.g. subject is presented with a simple geometric figure for 5 s, which is then removed and the subject is asked to draw it).

Successive Processing requires individuals to replicate a particular event or sequence of events. It contains Word Series, Sentence Repetition and Sentence Questions. In this subscale, the subject needs to understand or repeat auditory information that is presented in specific order. The Word Series is made up of monosyllabic words (e.g. dog, pen, and book) whose length ranges from 2 to 9 alphabets. The administrator reads them in a speed of 1 s/alphabet and asks the subject to repeat in the same order. The Sentence Repetition requires for a duplication of the auditory sentence that is made up of color items and with no actual meaning (e.g. red is black). The Sentence Questions are used to evaluate the subject’s understanding of the grammatical relations in the sentence by letting the subject to answer a question based on the stated sentence (e.g. ‘White is blue. What is blue?’ answer ‘white’).

Calculate raw scores in the 12 subtests separately, then convert them to scale scores. After that, add up the scale scores in each subscale and convert them to standardized scores.

### Statistical analysis

Continuous variables were shown as the mean value ± SD. Normal distribution of data was analyzed using the Kolmogorov–Smirnov normality test. Data with normal distribution were compared by Student’s t test and bonferroni correction. Those with unequal variance or without a normal distribution were analyzed by a Mann–Whitney rank sum test. Items of DN: CAS and FSIQ were compared by Student’s t test, and statistical analyses were carried out using statistical analysis software package SPSS19.0 with the level of significance at *p* < 0.05.

ROC curve was used to measure classification performance (sensitivity and specificity) for the DN: CAS and statistical analyses were carried out using statistical analysis software MedCalc15.8 with the level of significance at p < 0.05.

## Results

### Characteristics of study population

The general characteristics of the ADHD and control group are presented in Table 1; there were not significant differences of age, gender and grade between ADHD group and control group. Comparing to control group children with ADHD had significantly lower scores in FSIQ.

**Table 1:** The general characteristics of Study Population

Variables	ADHD	Control	*P* valve
Age(years)	9.1 ± 2.1	9.3 ± 1.5	0.299
Gender(n)			0.755
Boys	112	114	
Girls	23	26	
Grade(n)			0.217
First grade	17	12	
Second grade	31	20	
Junior class	24	31	
Fourth grade	20	31	
Fifth grade	20	25	
Six grade	23	25	
FSIQ	98.33 ± 9.5	105.58 ± 14.9	0.000

#### The DN:CAS assessment of the ADHD and control group

In this study, children with ADHD had significantly lower scores in these subtests compared to control group: Planning (*T* = − 10.260, *p* < 0.01), Simultaneous (*T* = − 2.406, *p* < 0.05), Attention (*T* = − 7.075, *p* < 0.01) of DN:CAS, while the total scores were also statically lower in ADHD group compared to controls (*T* = − 7.284, *p* < 0.01). Compared with control group, ADHD children had worse performance in Matching Numbers (*T* = − 8.22, *p* < 0.01), Planned Codes (*T* = − 5.779, *p* < 0.01), Planned Connections (*T* = − 9.124, *p* < 0.01) of Planning; Verbal-Spatial Relations (*T* = − 2.278, *p* < 0.01) of Simultaneous and Expressive Attention (*T* = − 5.146, *p* < 0.01); and Number Detection (*T* = − 6.608,*p* < 0.01), Receptive Attention (*T* = − 4.059,*p* < 0.01) (Table 2). The results showed that there were more cognitive deficits in ADHD group than in healthy subjects, especially in relation to planning and attention of DN: CAS.

**Table 2:** DN: CAS full scale and subscale means and standard deviations for ADHD groups and Control groups

CAS Subscales	ADHD		Control		t	*P* valve
	M	SD	M	SD		
Planning	23.19	5.15	29.59	5.19	−10.260	0.000
Matching Numbers	8.42	2.34	10.90	2.65	−8.218	0.000
Planned Codes	7.36	2.02	8.93	2.46	−5.779	0.000
Planned Connections	7.41	2.18	9.76	2.09	−9.124	0.000
Simultaneous	34.92	6.05	36.59	5.49	−2.406	0.017
Nonverbal Matrices	12.06	2.91	12.18	2.53	−0.363	0.717
Verbal-Spatial Relations	10.56	2.55	11.42	2.61	−2.785	0.006
Figure Memory	12.30	3.04	12.99	3.56	−1.725	0.086
Attention	25.73	5.52	30.38	5.37	−7.075	0.000
Expressive Attention	8.52	2.37	10.12	2.77	−5.146	0.000
Number Detection	8.50	1.98	10.08	1.99	−6.608	0.000
Receptive Attention	8.72	3.13	10.18	2.84	−4.059	0.001
Successive	32.47	6.25	33.44	5.32	−1.387	0.167
Word Series	16.27	2.73	16.81	2.63	−1.650	0.100
Sentence Repetition	7.08	2.19	7.48	1.85	−1.632	0.104
Sentence Questions	9.11	3.09	9.15	2.84	−0.109	0.913
Full Scale	116.30	16.17	129.99	14.99	−7.284	0.000

### ROC analyses

**Fig. 1:**
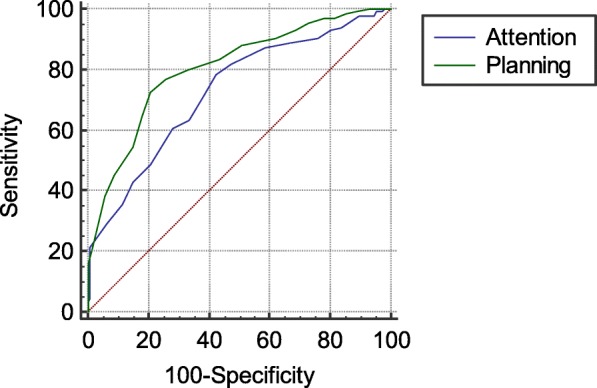
The ROC Curve of the Planning and the Attention to diagnostic the ADHD ROC analysis indicated that Planning and Attention had good classification accuracy with AUCs of 0.808 (95% CI: 0.756–0.853, *p* < 0.01) and 0.730 (95% CI: 0.673–0.782, *p* < 0.01) respectively

Since our results revealed an association between planning and attention deficit and ADHD assessment by DN:CAS, we aimed to investigate the sensitivity and specificity of planning and attention assessment in diagnosing ADHD. The ROC analysis indicated that Planning and Attention assessment had good classification accuracy in ADHD diagnose with AUCs of 0.808 (95%CI: 0.756–0.853, *p* < 0.01) and 0.730 (95%CI: 0.673–0.782, *p* < 0.01) respectively. The cutoff point was drawn from the curve for Planning 25-point and for Attention 29-point for diagnostic measures (Table 3 and Fig. 1). Positive predictive value and negative predictive values for Planning were 72.6 and 79.3%; for Attention were 78.5 and 57.9%, respectively.

**Table 3:** ROC curves and other parameters of Planning and Attention

Test Variables	Area	*P* Value	95%CI	
			Lower	Upper
Planning	0.808	0.0301	0.756	0.853
Attention	0.730	0.0260	0.673	0.782

## Discussion

The diagnosis of ADHD is mainly based on three categories of clinical symptoms, i.e. attention deficit, hyperactivity and impulsivity. Many scholars have tried to find an effective tool for diagnosis of ADHD, and from 1998, continuous performance tests (CPT) have been widely used in the diagnosis of ADHD. The CPT is used to assess attention and control ability [7, 8, 19]. Meanwhile, Go/No Go task is another test tool commonly used for ADHD diagnosis, which focuses on executive function but without assessing other cognitive functions [10]. Thus, the use of these tools has certain limitations in diagnosing ADHD and in evaluating the cognitive deficit in ADHD. The DN: CAS, a well-admissive theory-driven assessment kit consisting of four independent factors, which include Planning, Attention, Simultaneous and Successive processing in the PASS model, have been used to assess neurodevelopmental disorder in 5–17 year old children [15, 20]. To the best of our knowledge, the DN: CAS is the most comprehensive tool for assessment of cognitive processes, which is why we aimed to explore the association between ADHD diagnosis and the DN: CAS assessment.

Our results demonstrated that the ADHD gender incidence was 4.9 (112 boys): 1(23 girls), which was consistent with previous studies [1]. We found that ADHD children performed significantly worse in FSIQ and Planning including Matching Numbers, Planned Codes, Planned Connections; and Attention including Expressive Attention, Number Detection and Receptive Attention, which was consistent with the majority of recent researches [16, 20, 21]. According to PASS theory, Planning represents the ability to perform decision-making, selection and implementation, thanks to the corresponding brain regions that are located in frontal cortex [13, 22]. We found that ADHD subjects exhibited Planning defects in DN: CAS assessment, which was stated as EF impairment in Barkley’s theoretical model [6]. While Attention subtests require a sustained focus for identification of a target stimulus, our study revealed the ADHD children had a weaker performance in the Attention compared to healthy children. In addition, compared with control subjects, ADHD children scored worse in Verbal-Spatial Relations of Simultaneous, which was inconsistent with previous studies [14, 16]. According to PASS theory, Simultaneous is a cognitive process by which the individual integrates separate stimuli into a single group, and Simultaneous requires the children to understand logical-grammatical relationships [20]. Our results indicated that ADHD subjects might have deficit in Simultaneous processing of Verbal-Spatial reasoning.

Since the ADHD children appeared to have obvious defects in DN: CAS assessment, especially in planning and attention, ROC analysis was applied to evaluate the sensitivity and specificity of planning and attention assessment in diagnosing ADHD. The AUC of Planning was 0.808 when the cut-off point was set at 25-points, where the sensitivity was 72.6% and specificity was 79.3%; the AUC of Attention was 0.730, when the cut-off point was set at 29-points, where the sensitivity was 78.5% and specificity was 57.9%. The obtained results indicated that the planning and attention assessment of DN: CAS had high sensitivity and specificity in diagnosing ADHD, which suggested that DN: CAS might be an effective tool for diagnosing ADHD, especially in a clinical setting. This study also provided a useful indication for the treatment of ADHD by using remedial programmers; their specific focus will be the amelioration of EF deficit.

### Limitation

This study had some limitations. First, selection bias might exist in enrollment; Secondly, we recruited the ADHD cases without any comorbidities in order to reduce the possible bias from study subject’s stratification, we need to enlarge the study population of ADHD in the future; thirdly, larger sample needed to be carried out to verify the diagnostic value of DN:CAS in ADHD diagnosis.

## Conclusion

The planning and attention assessment of DN: CAS had highly sensitivity and specificity in diagnosing ADHD, which was encouraged that DN: CAS might be an effective tool in diagnosing ADHD.

## Abbreviations

ADHD: attention deficit hyperactivity disorder
APA: American Psychiatric Association criteria
CPT: continuous performance tests
DN: CAS: Das-Naglieri cognitive assessment system
DSM-5: Diagnostic and statistical manual of mental disorders 5th edition
FSIQ: Full Scale IQ
PASS: Planning, Attention, Simultaneous, and Successive Theory
